# [D-Ala2, D-Leu5]-enkephalin (DADLE) provides protection against myocardial ischemia reperfusion injury by inhibiting Wnt/β-Catenin pathway

**DOI:** 10.1186/s12872-024-03790-6

**Published:** 2024-02-19

**Authors:** Linwen Liu, Yawu Sun, Yang Wang, Jun Xin, Wei Chen

**Affiliations:** 1 Department of Cardiology, Shanghai Fourth People’s Hospital Affiliated to Tongji University, 1279 Sanmen Road, Hongkou District, Shanghai, 200434 China; 2 Department of Pathology, Shanghai Fourth People’s Hospital Affiliated to Tongji University, Shanghai, 200434 China; 3 Department of Ultrasonics, Shanghai Fourth People’s Hospital Affiliated to Tongji University, Shanghai, 200434 China

**Keywords:** [D-Ala2, D-Leu5]-enkephalin (DADLE), Myocardial ischemia reperfusion injury, Wnt/β-Catenin signaling pathway

## Abstract

**Background:**

Acute myocardial infarction is one of the leading causes of death worldwide. Myocardial ischemia reperfusion (MI/R) injury occurs immediately after the coronary reperfusion and aggravates myocardial ischemia. Whether the Wnt/β-Catenin pathway is involved in the protection against MI/R injury by DADLE has not been evaluated. Therefore, the present study aimed to investigate the protective effect of DADLE against MI/R injury in a mouse model and to further explore the association between DADLE and the Wnt/β-Catenin pathway.

**Methods:**

Forty-four mice were randomly allocated to four groups: Group Control (PBS Control), Group D 0.25 (DADLE 0.25 mg/kg), Group D 0.5 (DADLE 0.5 mg/kg), and Group Sham. In the control and DADLE groups, myocardial ischemia injury was induced by occluding the left anterior descending coronary artery (LAD) for 45 min. PBS and DADLE were administrated, respectively, 5 min before reperfusion. The sham group did not go through LAD occlusion. 24 h after reperfusion, functions of the left ventricle were assessed through echocardiography. Myocardial injury was evaluated using TTC double-staining and HE staining. Levels of myocardial enzymes, including CK-MB and LDH, in the serum were determined using ELISA kits. Expression of caspase-3, TCF4, Wnt3a, and β-Catenin was evaluated using the Western blot assay.

**Results:**

The infarct area was significantly smaller in the DADLE groups than in the control group (*P* < 0.01). The histopathology score and serum levels of myocardial enzymes were significantly lower in the DADLE groups than in the control group (*P* < 0.01). DADLE significantly improved functions of the left ventricle (*P* < 0.01), decreased expression of caspase-3 (*P* < 0.01), TCF4 (*P* < 0.01), Wnt3a (*P* < 0.05), and β-Catenin (*P* < 0.01) compared with PBS.

**Conclusions:**

The present study showed that DADLE protected the myocardium from MI/R through suppressing the expression of caspase-3, TCF4, Wnt3a, and β-Catenin and consequently improving functions of the left ventricle in I/R model mice. The TCF4/Wnt/β-Catenin signaling pathway might become a therapeutic target for MI/R treatment.

**Supplementary Information:**

The online version contains supplementary material available at 10.1186/s12872-024-03790-6.

## Introduction

Coronary heart diseases (CAD), the most common cardiovascular condition, have a high morbidity and mortality rate. Acute myocardial infarction is the main cause of death among CAD patients worldwide [1, 2]. Current myocardial reperfusion therapies, represented by percutaneous transluminal coronary intervention (PCI) and coronary artery bypass grafting (CABG), play a vital role in salvaging the ischemic myocardium from further necrosis. However, myocardial ischemia reperfusion (MI/R) injury which occurs immediately after the coronary reperfusion accelerates necrocytosis and exacerbates ischemic injury [3, 4]. Raising the tolerance to MI/R injury is important for improving the clinical efficacy of currently available treatments. Although there are a large number of studies aiming at preventing the progression of MI/R injury, only a minority of them have succeeded. As drugs have advantages in administration, it has gradually become a research hotspot in the field of organ protection in recent years.

DADLE is a synthetic delta-opioid receptor (DOR) agonist that can induce hibernation, protect peripheral organs, and promote cell survival. Accumulating evidence has revealed that DOR agonists can ameliorate I/R injuries of multiple organs [5–7]. A recent study reported that DOR agonists attenuated myocardial reperfusion injury, suggestive of a postconditioning effect [8]. The majority of previous studies on MI/R were conducted on in vitro experimental models or in vivo models with a short reperfusion time, such as 2 h. In the present study, the reperfusion time was extended to 24 h after 45-min ischemia in mice to observe the impact of DADLE on changes of cardiac functions and myocardial injury.

Furthermore, the mechanism of DADLE in alleviating MI/R injury still remains unknown. Our previous study showed that DADLE down-regulated the NF-κB signaling pathway [9]. Another study displayed that NF-κB interacted with the Wnt signaling pathway in cardiomyocytes [10]. It is known that the Wnt/β-Catenin signaling pathway plays an important role in cell development and differentiation, especially in cell apoptosis [11]. Whether the Wnt/β-Catenin pathway is involved in the protection against MI/R injury by DADLE has not been evaluated. Therefore, the present study aimed to investigate the protective effect of DADLE against MI/R injury in a mouse model and to further explore the association between DADLE and the Wnt/β-Catenin pathway.

## Materials and methods

### Reagents

DADLE was purchased from Abcam Biotechnology (Cambridge, MA, USA). TTC and Evans blue were obtained from Sigma Chemical Co. (Louis, MO, USA). Antibodies against TCF4, Wnt3a, β-Catenin, and caspase-3 were purchased from Abcam Biotechnology. Secondary antibodies were purchased from LI-COR Technology (Lincoln, NE, USA). Creatine kinase-MB isoenzyme (CK-MB) and lactate dehydrogenase (LDH) kits were obtained from Enzyme Linked Biotechnology (Shanghai, China).

### Animals

Forty-four C57BL/6J mice, weighing 24–26 g, were purchased from Regen Biotechnology Ltd (Shanghai, China). They were placed in a 12 h light/dark cycle room at a constant temperature (23 ± 1 °C) and humidity (40−60%) with free access to food and water. These mice were raised under standard conditions for 1 week to adapt to the new environment. This study was performed according to the Guide for the Care and Use of Laboratory Animals, which was published by the US National Institutes of Health (National Institutes of Health Publication no. 85 − 23, revised 1996) and was approved by the Ethics Committee of Shanghai Fourth People’s Hospital (approval number: TjHBLAC-2020-39).

### Drug administration to different groups

**Fig. 1:**
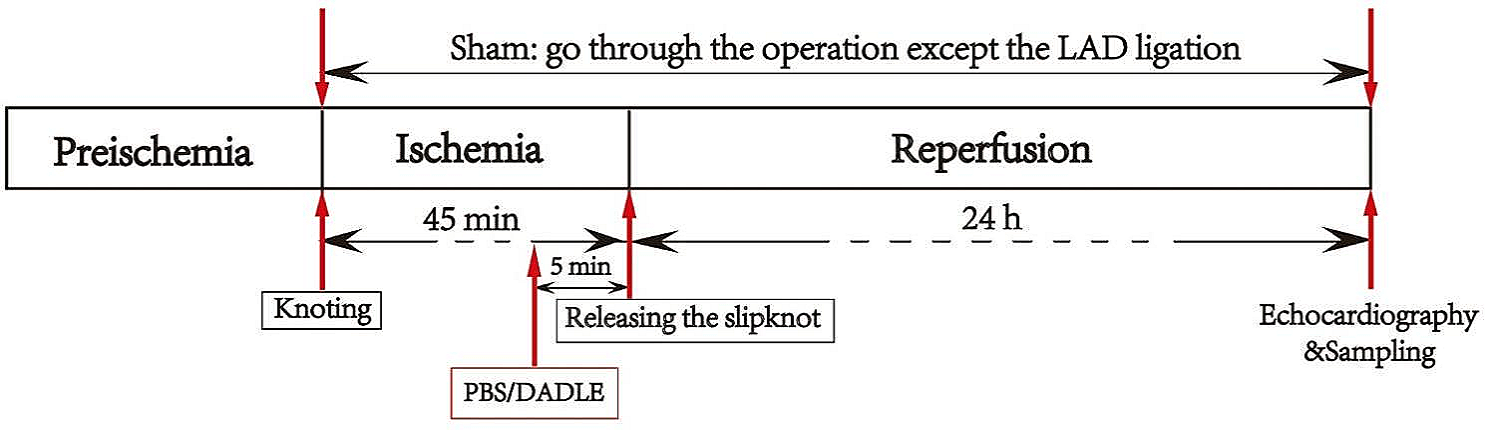
Illustration of the experimental design

Mice were randomly allocated to 4 groups: group control (PBS control, *n* = 12), group D 0.5 (DADLE 0.5 mg/kg, *n* = 15), group D 0.25 (DADLE, 0.25 mg/kg, *n* = 5), and group sham (*n* = 12). DADLE was dissolved in PBS. In the control and DADLE groups, PBS or DADLE at different doses were administrated intraperitoneally, respectively, during the ischemic period 5 min before reperfusion. In the sham group, an identical surgical procedure was performed without ligating the left anterior descending coronary artery (LAD). The sham and control groups received equal volumes of the PBS solution according to the same schedule (Fig. 1).

### Modeling of MI/R

In brief, the model of cardiac injury was induced and modified on the basis of the classical one reported by Gao et al. [12]. Mice were anesthetized with 2–5% isoflurane. Myocardial ischemia was induced by exposing the heart with a left thoracic incision, followed by making a slipknot (6–0 silk) around the LAD. In the meantime, changes of the electrocardiograph (ECG) were monitored. After 45 min of ischemia, the slipknot was released, and reperfusion was maintained for 24 h. Mice in the sham group received the same operation procedures except that LAD was not ligated. Tissue samples were collected 24 h following induction of MI/R.

### Measurement of the infarct size

LAD was ligated again after 24 h of ischemia–reperfusion treatment. 2% Evans blue was injected into the left ventricle. Then the heart was isolated, frozen at -80˚C for 30 s, sliced into 5 Sect. (2 mm) and incubated in 2% TTC for 15 min. The necrotic myocardium showed a pale color and the viable one appeared red. Non-ischemic area turned blue. The ischemic area (IA, pale) and the area at risk (AAR, pale + red) were photographed and measured by Image Pro Plus 6.0 (Media Cybernetics, USA). The IA / AAR (%) was calculated to evaluate changes of the infarct area.

### Echocardiography

24 h after reperfusion, echocardiography was performed under anesthesia using the Vevo2100 imaging system (VisualSonics Inc., Toronto, ON, Canada) with a 30-MHz probe by an experienced ultrasound doctor. M-mode images were recorded. Both the left ventricular end-systolic dimension (LVESD) and the left ventricular end-diastolic dimension (LVEDD) of mice were measured manually. The left ventricular ejection fraction (EF), fraction shortening (FS), the left ventricular end-diastolic volume (LVEDV) and the left ventricular end-systolic volume (LVESV) were calculated automatically by the ultrasound machine.

### Detection of activities of serum CK-MB and LDH

Blood samples were collected from the eyeballs of the mice and centrifuged at the due time. The supernatants were subsequently isolated. Activities of serum CK-MB and LDH were detected using ELISA assay kits according to instructions provided by the manufacturer.

### HE staining

Mice were sacrificed and their heart samples were excised and fixed in 10% phosphate-buffered formalin for 48 h, and subsequently embedded in paraffin. Serial slices were collected and stained with H&E for histological examination. Morphological changes were observed under a microscope.

The pathology of each heart slice was scored using the method developed by Yin et al. [13]: (0) zero; (1) one = focal myocyte injury; (2) two = small multifocal degeneration with inflammation to a minor degree, and the order of myocardial fibers is occasionally disorganized; (3) three = extensive myofibrillar degeneration and/or diffuse inflammation, in which cells are moderately damaged and rupture of myocardial fibers is observed, and (4) four = necrosis with diffuse inflammation: cells are extensively injured. Myocardial necrosis is accompanied by diffuse inflammation. The arrangement of myocardial fibers is disorganized. Six different visual fields were randomly selected for each slide and the pathology was scored. Analysis was conducted by two observers who were blind to the experimental procedures.

### Western blot analysis

Proteins from ischemic heart tissues were extracted and quantified with the BCA protein assay kit (Thermo Fisher Pierce, Rockford, USA). Protein samples of 20 µg were separated on the SDS -PAGE gel and transferred onto the poly vinylidene difluoride membrane. The membrane was then blocked with 3% bovine serum albumin for 2 h at room temperature, followed by incubation in the primary antibody solution (caspase-3, TCF4, Wnt3a, and β-Catenin, respectively) at 4℃ for overnight. Then, the membrane was washed with PBS and incubated in the horseradish peroxidase-conjugated secondary antibody solution for 1 h at room temperature. The enhanced chemiluminescence (ECL) kit was used to visualize the blot bands. The results were imaged using Image Lab (Bio–Rad, Hercules, USA). The optical density of protein bands was analysed using Image J.

### Immunohistochemical staining

After dewaxing paraffin-embedded sections and antigen retrieval, sections were blocked with 0.3% Triton X-100 and 10% bovine serum albumin for 1 h, followed by incubation in the anti-Wnt3a antibody solution at 4℃ for overnight. After washing with PBS, these sections were incubated with the Alexa Fluor 488 conjugated goat anti-mouse secondary antibody solution for 1 h, and then counterstained with a propidium iodide solution for 5 min in the dark. After washing with PBS, these sections were coverslipped and sealed with a nail oil. Sections were visualized under a fluorescence microscope.

### Statistical analysis

Statistical analyses of data were performed using SPSS 26. Student’s *t* test or One-way analysis of variance followed by Tukey’s multiple comparisons was adopted for continuous variables. Ultrasonic parameters were expressed as median (interquartile range). Other data in the figures were presented as mean ± SD. The difference was considered statistically significant if *P* value was < 0.05.

## Results

### Evaluation of myocardial injuries

**Fig. 2:**
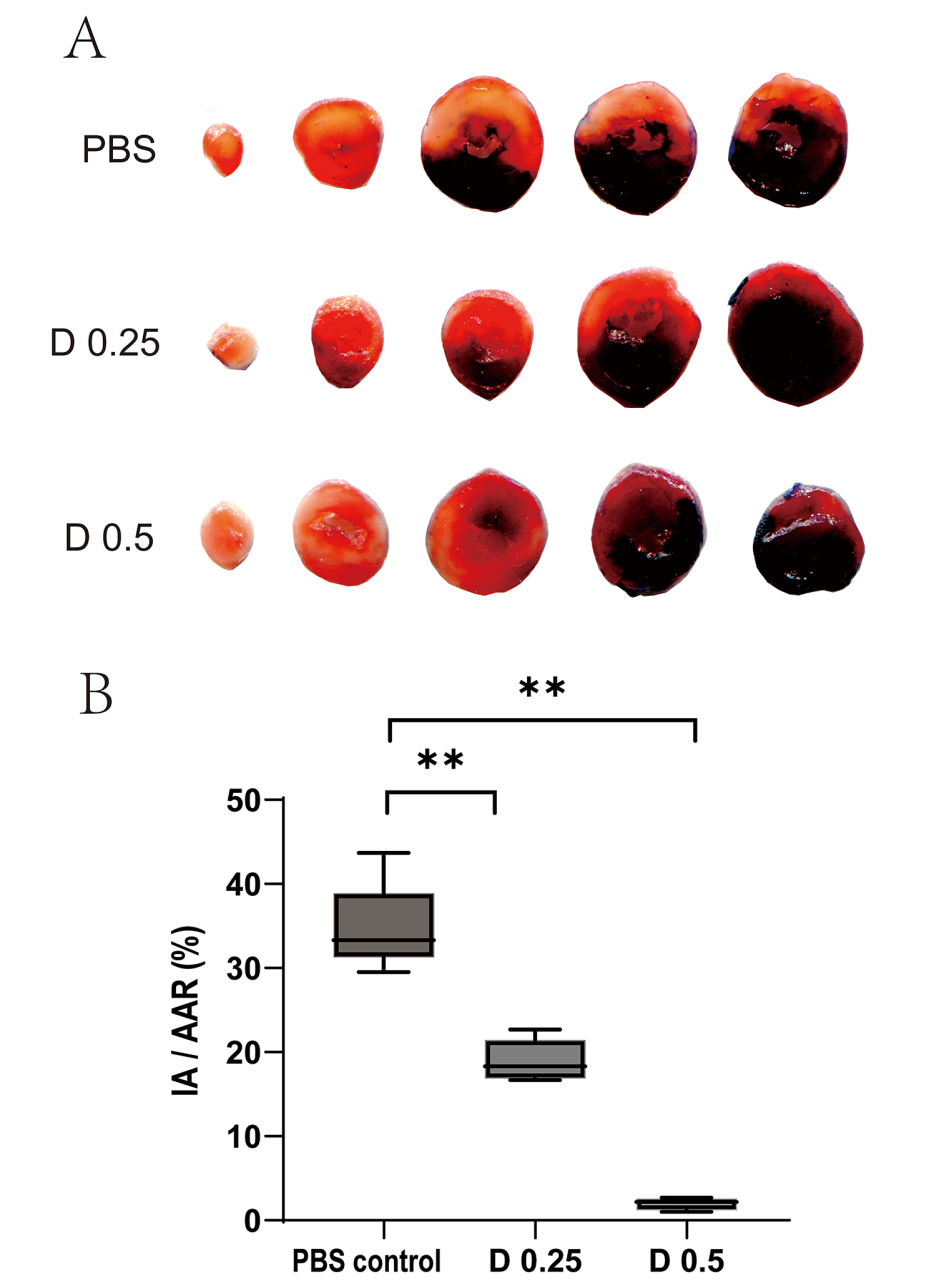
DADLE significantly reduced the myocardial infarct size in mice. **A**. Representative images of TTC/Evans blue double staining in the PBS, D 0.25, and D 0.5 groups 24 h after reperfusion. **B**. The IA / AAR (%). Data were presented by mean ± SD, **p < 0.01 vs. PBS group. D 0.25: DADLE 0.25 mg/kg; D 0.5: DADLE 0.5 mg/kg. *n* = 5 for each group

To evaluate the effect of DADLE on myocardial injury, Evans blue/TTC double-staining was used to measure the infarct size. As shown in Fig. 2, both DADLE 0.25 mg/kg and 0.5 mg/kg significantly reduced the infarct area compared with PBS (*P* < 0.01). The IR/AAR (%) was reduced by 17% in the D 0.25 group and 33% in the D 0.5 group compared with that of the control group (Fig. 2B). DADLE 0.5 mg/kg was more potent than DADLE 0.25 mg/kg in reducing the infarct size. As a result, we chose the dose of 0.5 mg/kg to further verify the protective effect of DADLE on myocardial injury.

**Fig. 3:**
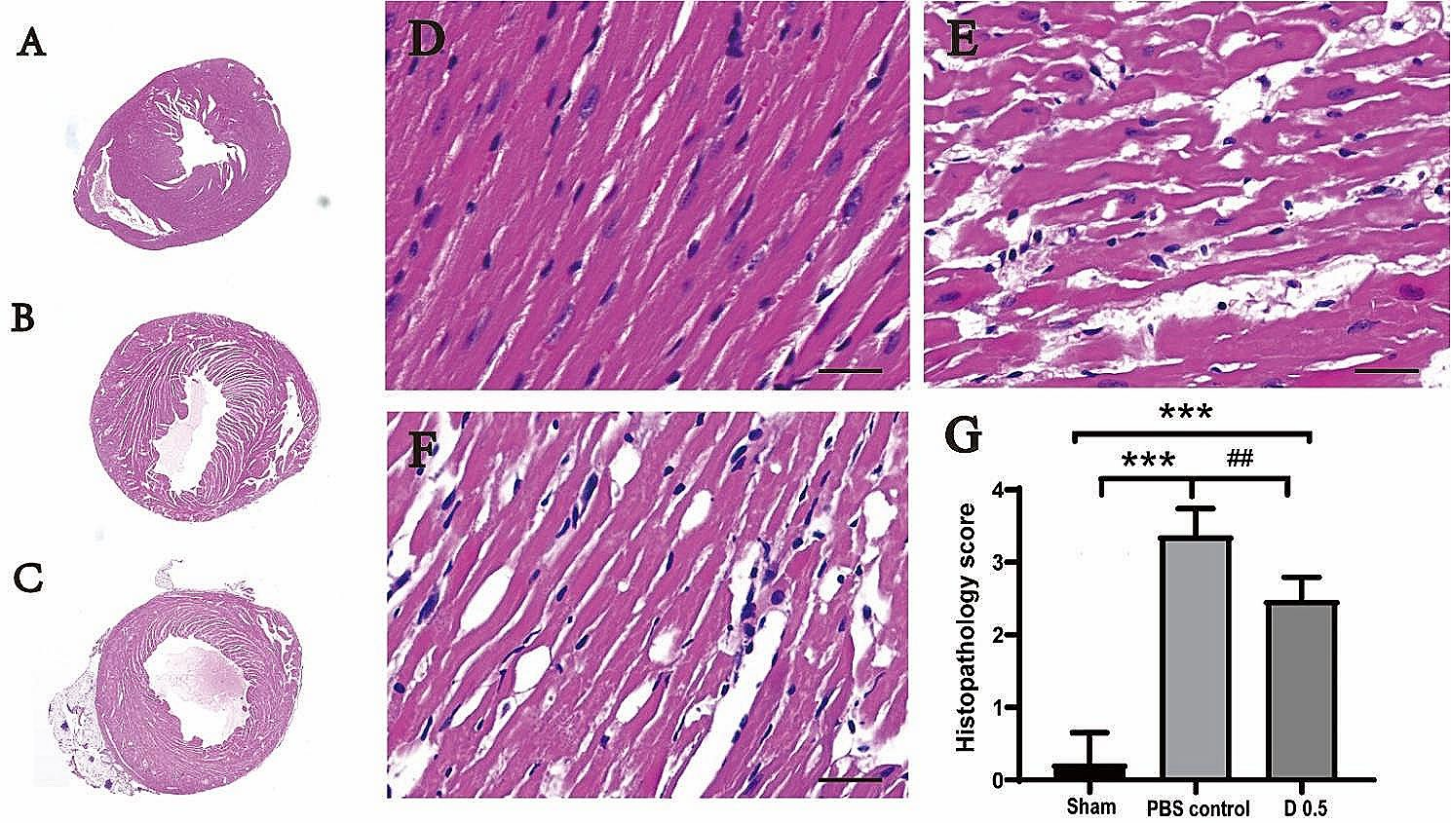
DADLE decreased the cardiac histopathology score in MI/R mice. (**A-C**) Representative HE images of myocardial cells at a low magnification. (**D-F**) Representative HE images of myocardial cells in the left ventricular anterior wall at a high magnification. (**A, D**) The sham group. (**B, E**) The control group. (**C, F**) The DADLE group. Scale bar = 20 μm. (**G**) Comparison of the histopathology scores between groups. ***P < 0.001 vs. Sham. ##*P* < 0.01 vs. Control. D 0.5: DADLE 0.5 mg/kg. *n* = 3 for each group

Representative photomicrographs of the myocardium at a low magnification were displayed in Fig. 3A-C. Representative HE images of myocardial cells in the left ventricular anterior wall at a high magnification were shown in Fig. 3D-F. The histopathology scores of the control group and the DADLE 0.5 mg/kg group were 3.4 and 2.5, respectively, which were significantly higher than that in the sham group sham (count approaching 0, *P* < 0.001, Fig. 3G). Similarly, the histopathology score of the DADLE group was significantly lower than that of the PBS control group (*P* < 0.01). To conclude, DADLE protected myocardial cells against I/R injuries at the dose of 0.5 mg/kg.

**Fig. 4:**
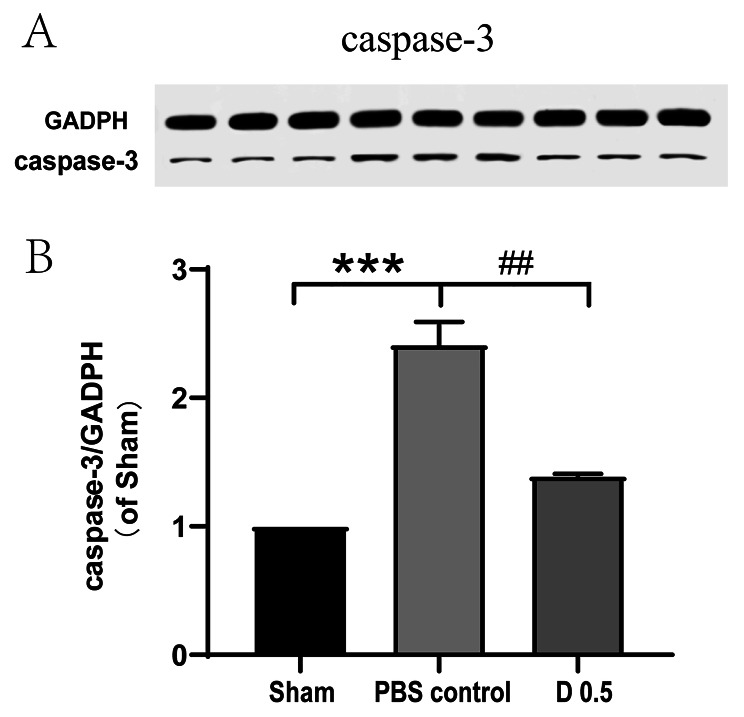
DADLE decreased apoptosis of myocardial cells. Levels of caspase-3 were detected using Western blot analysis. (**A**) expression of caspase-3 on westernblot. The first 3 lanes were from the sham group, the middle 3 lanes were from the control group, and the last 3 lanes were from the DADLE group. (**B**) Western blot analysis of caspase-3 expression. Results were presented as mean ± SD. ***P < 0.001 vs. Sham. ##*P* < 0.01 vs. Control. D 0.5: DADLE 0.5 mg/kg. *n* = 3 for each group

To evaluate the effect of DADLE on cardiac apoptosis, expression of caspase-3 in the ischemia region was detected. Western blot (Fig. 4) showed that the level of caspase-3 was significantly higher in the control group than in sham group, indicating the presence of cardiac injury (*P* < 0.001). DADLE remarkably decreased caspase-3 expression compared with PBS, which suggests the anti-apoptosis effect of DADLE (*P* < 0.01).

**Fig. 5:**
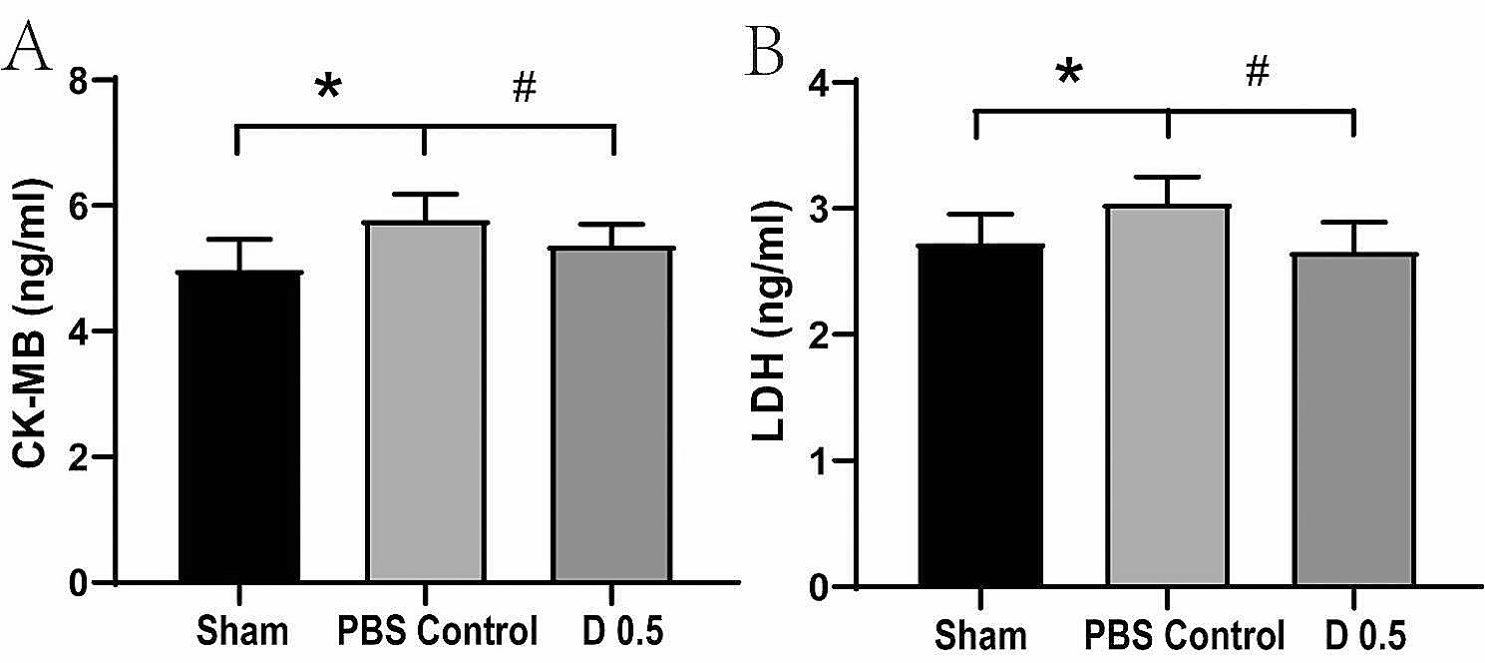
DADLE decreased levels of CK-MB and LDH as shown by the ELISA assay. (**A**) CK-MB activity. (**B**) LDH activity. *P < 0.05 vs. Sham. #*P* < 0.05 vs. Control. D 0.5: DADLE 0.5 mg/kg. *n* = 10 for each group

LDH and CK-MB are two commonly used indicators of myocardial injury. In the present study, content of CK-MB (Fig. 5A) and LDH (Fig. 5B) in the heart tissue were evaluated using ELISA kits. Compared with the sham group, the serum level of CK-MB was significantly increased in the control group 24 h after reperfusion (*P* < 0.05). DADLE significantly attenuated this increase (*P* < 0.05) bringing the level of CK-MB to a similar level of the sham group. Similar phenomenon was observed for LDH. The level of LDH was significantly decreased after the administration of DADLE compared with that of the control group (*P* < 0.05). These results demonstrated that DADLE alleviated MI/R-induced injury.

### Assessment of cardiac functions

**Fig. 6:**
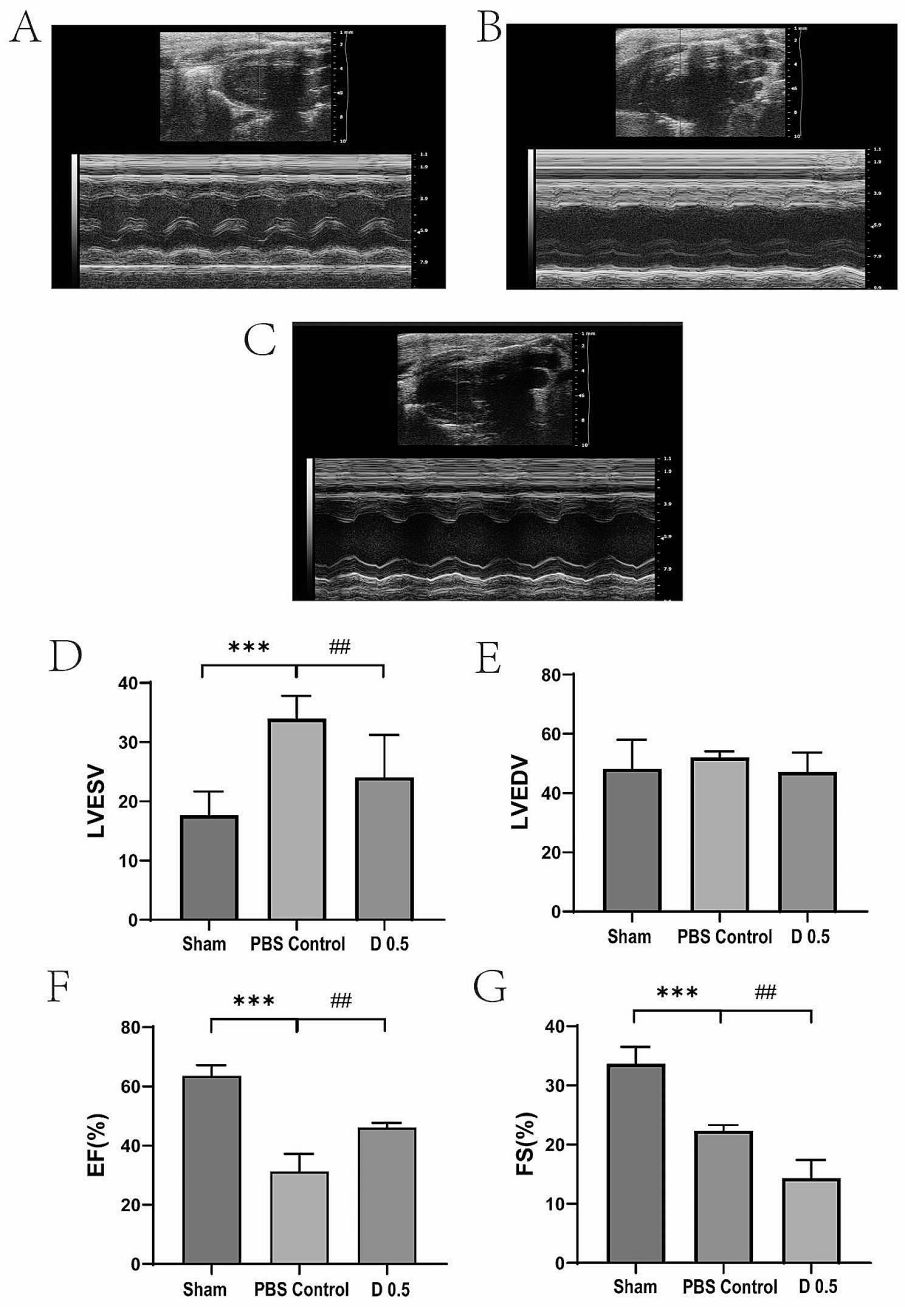
DADLE improved functions of the left ventricle in the mouse model of MI/R. (**A-C**) Representative ultrasound patterns of the three groups 24 h after reperfusion. (**A**) The sham group. (**B**) The control group. (**C**) The DADLE group. (**D**) Comparison of the LVESV between groups. (E) Comparison of the LVEDV between groups. (**F**) Comparison of the EF between groups. (**G**) Comparison of the FS between groups. The error bars showed the median values. D 0.5: DADLE 0.5 mg/kg. ***P < 0.001 vs. Sham. ##*P* < 0.01 vs. Control. *n* = 10 for each group

To investigate the effect of DADLE on cardiac functions, the M mode echocardiogram was used to measure cardiac parameters. Representative ultrasound patterns at the moment of 24 h after reperfusion were shown in Fig. 6A-C.

As demonstrated in Fig. 6D, the LVESV was significantly increased in the control group compared with that of the sham group (*P* < 0.001). DADLE (0.5 mg/kg) remarkably reversed this increase in the mouse model (*P* < 0.01). No significant difference in LVEDV was observed between groups. In other words, the LVEDV was not affected by either MI/R or DADLE (Fig. 6E). After ischemia, EF and FS significantly reduced in control group compared with sham group (Fig. 6E-G).

Interestingly, DADLE significantly improved the EF and the FS in the mouse model (the control group) (*P* < 0.01). These results indicated that DADLE can improve functions of the left ventricle in MI/R mice.

### Exploration of mechanisms underlying the protective effect of DADLE

**Fig. 7:**
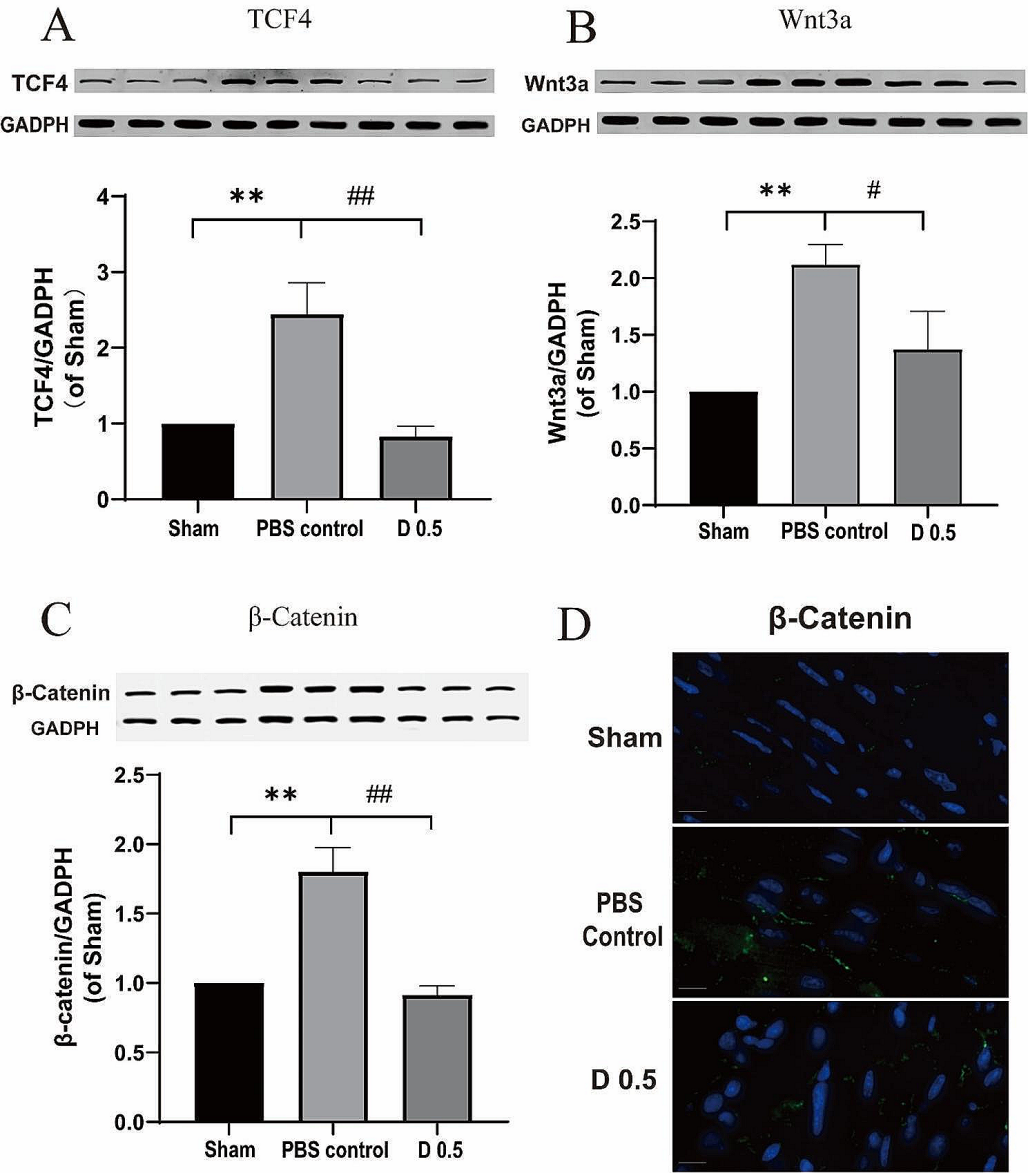
DADLE suppressed expression of proteins of the Wnt3a/β-Catenin pathway. (**A**) Expression of TCF4, (**B**) Wnt3a, and (**C**) β-Catenin on Western blot. (**D**) Immunofluorescence staining showing the expression of β-Catenin (green: β-Catenin; blue: DAPI; scale bar = 10 μm). D 0.5: DADLE 0.5 mg/kg. **P < 0.01 vs. Sham. #*P* < 0.05 vs. Control. ##*P* < 0.01 vs. Control. *n* = 3 for each group

To examine whether the Wnt/β-Catenin signaling pathway is involved in the cardioprotective effects of DADLE, the expression levels of TCF4, Wnt3a, and β-Catenin were detected using Western blot. As shown in Fig. 7A, the expression of TCF4 was dramatically increased in the control group compared with that of the sham group (*P* < 0.01). DADLE 0.5 mg/kg significantly reversed the expression level of TCF4 in the mouse model (*P* < 0.01). As shown in Fig. 7B and C, the expression levels of Wnt3a and β-Catenin were significantly increased in the control group compared with those of the sham group (*P* < 0.01). DADLE 0.5 mg/kg treatment significantly reversed the expression levels of Wnt3a (*P* < 0.05) and β-Catenin (*P* < 0.01) in the mouse model. Immunofluorescence staining showed similar results to Western blot (Fig. 7D), indicating that DADLE can significantly reduce the expression of β-Catenin in the mouse model.

## Discussion

The present study investigated the cardioprotective effect of DADLE, a δ opioid receptor agonist, on the MI/R injury mouse model. It was found that DADLE significantly decreased the infarct area and serum levels of CK-MB and LDH, and improved functions of the left ventricle in the MI/R injury mouse model, specifically the systolic contraction reflected by the LVESV, but not the LVEDV. The latter may be explained by the passive dilation of the ventricle due to the incoming blood flow. In the meantime, DADLE significantly decreased expression levels of TCF4, Wnt3a, and β-Catenin. These suggest that DADLE may alleviate I/R injury of the myocardium through suppressing the Wnt3a/β-Catenin pathway.

Opioid receptors are mainly categorized into κ (KOR), µ (MOR), and δ (DOR) subtypes, all of which belong to G protein-coupled receptors. DORs are widely distributed in the central nervous system, peripheral nervous system and the cardiovascular system [14]. In our previous study, we found that DADLE, a synthetic DOR agonist, played a protective role in cerebral and spinal cord I/R injury [9, 15]. Multiple studies have demonstrated that the expression of DORs can be detected in the heart tissue of animals and humans [16–18]. An in vitro study has demonstrated that DADLE, when administered during the early reoxygenation period, protected human myocardium from hypoxia–reoxygenation injury [16]. Delta opioid-induced preconditioning enhanced cardiac adaption to stress after 30-min ischemia and 2 h- reperfusion in mice [19]. Our findings are consistent with those of previous studies in that DOR agonists notably attenuated MI/R injury in mice. The beneficial effect of the DOR agonist in our study was evidenced by the reduced myocardial infarct size as shown in TTC-Evans blue staining, decreased serum levels of myocardial enzymes, decreased apoptosis of myocardial cells and decreased expression levels of caspase-3, TCF4, Wnt3a, and β-Catenin, and improved functions of the left ventricle in the mouse model of MI/R injury. Our results coincide with those of the study by Fuardo et al. [16]. It is reasonable to conclude that DOR agonists are efficacious in protecting the myocardium from ischemia-reperfusion injury.

However, there are also studies reporting conflicting results in recent years. For instance, a study found that DOR activation attenuated the contracting capacity of human heart muscles in vitro after ischemia and reperfusion [20]. In a novel in vitro model of engineered human heart tissue, DADLE showed no protective effect, which is consistent with the absence of DOR expression in the heart tissue [21]. The vast majority of studies on MI/R injury are conducted in vitro or on animals with a short reperfusion time, such as 2 h. In our animal model, the reperfusion time was extended to 24 h after 45-min ischemia, which facilitated the investigation of the long-term effect of therapeutics targeting the remodeling of the heart tissue. The discrepancy between our study and other previous ones may be attributed to the use of distinct experimental models, in which reperfusion time, dosage of the drug, administration time, and even experimental subjects are different. In addition, DADLE may take its effect through complex mechanisms which are different from those discovered in vitro. It has been reported that cardiac MORs were substantially up-regulated during heart failure which augmented cardioprotection against I/R injury [22]. Activation of central opioid receptors by morphine increases the resistance of the heart to I/R injury [23]. Therefore, it can be speculated that expression of DORs may also be impacted by pathological conditions and DOR agonists may take effect through stimulating central opioid receptors rather than solely activating DORs in the heart. More scientific research is needed to explore the detailed mechanism.

After MI/R, a complex cascade of pathophysiologic processes take place and encroach the neighboring cardiomyocytes. These processes include inflammatory responses to ischemia, oxidative stress and activation of apoptosis pathways. The Wnt/β-Catenin signaling pathway plays a vital role in regulating apoptosis. Activation of the Wnt cascade leads to translocation of β-Catenin to the nucleus and stimulates a family of transcription factors including TCF4, which motivate the downstream target genes related to cell apoptosis [11]. After myocardial infarction, the Wnt signaling pathway can be constantly activated, which reduces apoptosis and accelerates ventricular remodelling [24]. Yang et al. reported that activating the Wnt/β-catenin signaling pathway by an miR-148b inhibitor improved myocardial cell survival after MI/R [25]. Xie et al. reported that cardiomogen ameliorated cardiac dysfunction by decreasing β-catenin after myocardial infarction [26]. Not suprisingly, there are controversies about the regulation of Wnt signaling pathways in cardiac injury. We speculate that upregulation of Wnt/β-catenin pathway reduces apotosis in early stage of ischemia/reperfusion, wheras inhibition of Wnt/β- catenin can alleviate cardiac remodeling in the late stage of reperfusion, attenuating myocardial injury. We confirmed the effect of DADLE on the Wnt/β-Catenin signaling pathway in MI/R mice focusing on cardiac remodeling stage.

A study showed that opioid receptors can act through the Wnt signaling pathway. Wu et al. [27] reported that Wnt signaling contributed to withdrawal symptoms from prolonged MOR activity induced by chronic morphine exposure. Currently, no research has investigated the relationship between DORs and the Wnt signaling pathway in MI/R injury models. For the first time, we experimentally verified that DADLE remarkably mitigated MI/R injury in association with inhibition of the Wnt/β-Catenin signaling pathway.

As mentioned before, TCF7L2, also named TCF4, mediates canonic Wnt/β-Catenin signaling and c-Myc upregulation during abnormal cardiac remodeling in heart failure [28]. Suppresion of β-Catenin activation results in a downregulation of TCF7L2 protein and rescues cardiac function [29]. In line with previous studies, expression of TCF4 was decreased and functions of the left ventricle were reversed by DADLE in our research.

Our study also has its own limitations. Only data at 24 h after reperfusion were observed. Data from more time points may contribute to determine the pharmacokinetics of DADLE. Certainly, it is necessary to validate the dose-response curve and find the best dosage for translation.

In summary, our study has clearly illustrated that postconditioning with DADLE alleviated MI/R injury in mice. Suppressing the activation of the TCF4/Wnt/β-Catenin signaling pathway could be one of the underlying mechanisms. DADLE may be an effective therapeutics to prevent MI/R injury during cardiac surgery.

### Electronic supplementary material

Below is the link to the electronic supplementary material.

Supplementary Material 1

## Data Availability

The data for preparing this article will be available from the corresponding author upon reasonable request.

## Abbreviations

MI/R: Myocardial ischemia reperfusion
I/R: Ischemia reperfusion
LAD: Left anterior descending coronary artery
TTC: Triphenyltetrazolium chloride
HE: Hematoxylin-eosin
TCF4: T cell factor 4
EF: Left ventricular ejection fraction
FS: Fraction shortening
CAD: Coronary heart disease
DOR: Delta opioid receptor
CK-MB: Creatine kinase-MB isoenzyme
LDH: Lactate dehydrogenase
LVESD: Left ventricular end-systolic dimension
LVEDD: Left ventricular end-diastolic dimension
LVEDV: Left ventricular end diastolic volume
LVESV: Left ventricular end systolic volume
