# Supplementary figures and images for: [D-Ala2, D-Leu5]-enkephalin (DADLE) provides protection against myocardial ischemia reperfusion injury by inhibiting Wnt/β-Catenin pathway
Source: BMC Cardiovasc Disord. 2024 Feb 19;24:115. doi: 10.1186/s12872-024-03790-6 (PMC10877899; doi:10.1186/s12872-024-03790-6)

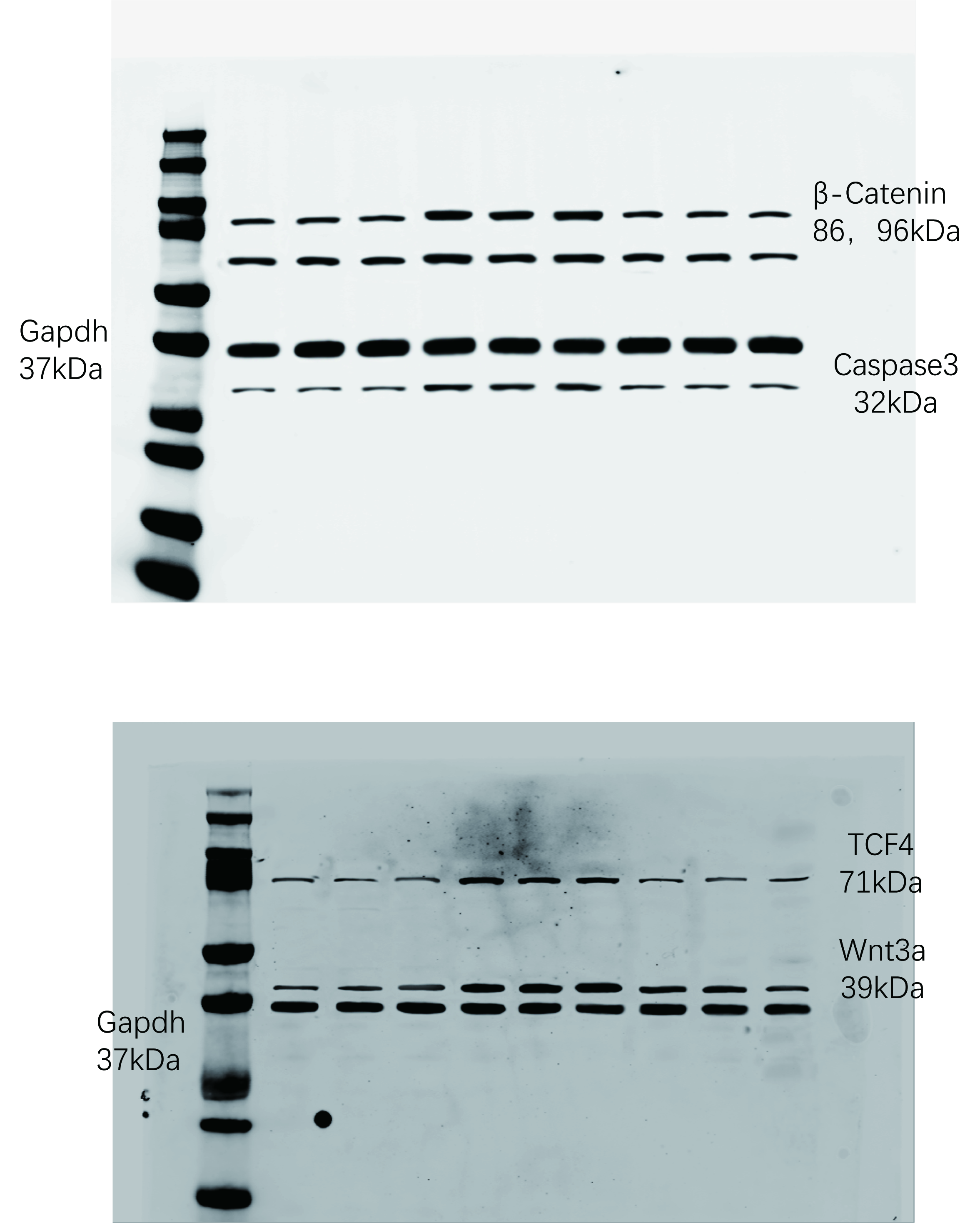

Supplement: Supplementary file 1 — Supplementary Material 1 [file 12872_2024_3790_MOESM1_ESM.tif]
